# Chemoradiation for cervical cancer treatment portends high risk of pelvic floor dysfunction

**DOI:** 10.1371/journal.pone.0234389

**Published:** 2020-06-12

**Authors:** Taís Pereira Miguel, Carla Elaine Laurienzo, Eliney Ferreira Faria, Almir José Sarri, Isabela Queiroz Castro, Renato José Affonso Júnior, Carlos Eduardo Mattos da Cunha Andrade, Marcelo de Andrade Vieira, Ricardo dos Reis

**Affiliations:** 1 Department of Physiotherapy, Barretos Cancer Hospital, São Paulo, Brazil; 2 Department of Urology, Barretos Cancer Hospital, São Paulo, Brazil; 3 Department of Epidemiology and Biostatistics, Barretos Cancer Hospital, São Paulo, Brazil; 4 Department of Radiation Theraphy, Barretos Cancer Hospital, São Paulo, Brazil; 5 Department of Gynecologic Oncology, Barretos Cancer Hospital, São Paulo, Brazil; University Medical Center Utrecht, NETHERLANDS

## Abstract

**Goal:**

To assess the impact of chemoradiation on pelvic floor (PF) muscle function after the treatment of cervical cancer (CC).

**Methods:**

We performed a prospective cohort study of women between the ages of 20 and 70 years old who had a diagnosis of CC. Patients were treated with chemoradiation at the Barretos Cancer Hospital (BCH), between August 2016 and July 2017. We performed three evaluations at different time points after chemoradiation treatment to compare changes in muscle function. Pelvic floor muscle function was assessed through perineometry (PNM) and surface electromyography (EMG) at the following time points: Pretreatment Moment 1 (M1): evaluated before chemoradiation; Moment 2 (M2): at the first follow-up medical visit (usually 3 to 4 months after treatment); and Moment 3 (M3): at the second follow-up medical visit (usually after 6 to 9 months after treatment). Mean vaginal squeeze pressure levels were determined by PNM and muscle electromyographic activity by EMG and the results were evaluated by Generalized Linear Model comparisons.

**Results:**

Forty-nine patients were evaluated at M1; 35 at M2; and 32 at M3, so that 32 patients had all three muscle evaluations performed. There was a statistically significant increase in the frequency of women with urgency urinary incontinence at the M2 evaluation time (41.9%), compared to pretreatment M1 (18.6%), p<0.001. The means of the vaginal squeeze pressures reduced through M1 to M3 in the phasic (M1: 17.7 mmHg; M3: 11.27mmHg) and tonic contractions (M1: 10.56 mmHg; M3: 7.52mmHg), *p* = 0.01 and *p* = 0.03 respectively. There was no difference in pelvic floor function in the three evaluations M1-M3, measured by EMG. The pelvic floor strength assessed by PMN and their interactions with anthropometric, parity and hormonal status variables, showed that a high body mass index (BMI) significantly influenced decreases in pelvic floor muscle function before and after treatment.

**Conclusion:**

These results show that chemoradiation causes reduction of muscle function of the pelvic floor, especially in the late phase after the end of treatment. Both the high BMI and urgent urinary incontinence symptoms were related to decreased muscle strength.

## Introduction

Cervical cancer (CC) is a significant public health problem being the fourth most common cause of cancer in women worldwide. The International Agency for Research on Cancer estimated that in 2018 there were 570,000 new cases of CC and 311,000 deaths [1]. Despite the high incidence, mortality rates for this neoplasia have decreased significantly in recent decades. This progress is due to both improvements in technologies used to treat the disease and the introduction of increased screening programs [1]. The primary treatment for advanced tumors is chemoradiation, which has been shown to reduce recurrence rates and increase survival [2,3]. External radiotherapy and brachytherapy can be curative in 30 to 60% of cases [4]. However, even though there have been considerable treatment gains through technological advances such as Intensity-modulated radiotherapy (IMRT), altered fractionation and the introduction of new drugs combination [5], an ongoing problem is that pelvic radiation can still compromise adjacent tissues and organs [3].

Radiotherapy is known to result in alterations of morphology and function of the female pelvic floor muscles (PFMs) [6]. The PFMs play an essential role in pelvic organ support, the maintenance of continence and are also associated with healthy sexual function [7–9].

In gynecologic neoplasms, radiotherapy can cause actinic lesions to the pelvic floor muscles [10], which are thought to play a role in the high prevalence of urinary incontinence, fecal incontinence and sexual disorders after radiotherapy [11–13]. It is known from women without a history of cancer that structural and functional changes in the pelvic floor muscles negatively impact their ability to generate force, contributing to dysfunction and incontinence [14]. There are few previous studies evaluating the effects of radiotherapy and/or chemoradiation on the pelvic floor function after treatment for gynecological cancer. Moreover, most studies have divergent results and methods of evaluation [6, 13, 15]. Noronha et al. evaluated 60 women after CC treatment and did not find any difference in the contraction capacity of the pelvic floor among women who exclusively underwent surgery, or had radiotherapy alone or just underwent chemoradiation. However, the measurement was performed by bidigital palpation, which is a less reliable method of evaluation [13]. On the other hand, studies by Yeoh et al. and Bernard et al. demonstrated a reduction in the function of the pelvic floor muscles in women after endometrial and cervical cancers. These alterations were demonstrated through a lower squeeze pressure assessed by manometry by the former, and through a lower maximum strength assessed by the dynamometry by the latter, both reliable methods for the assessment of pelvic floor muscles [6, 15].

Although the main focus of cancer management must be to treat the cancer itself, pelvic floor dysfunctions such as incontinence and sexual dysfunctions are prevalent after these treatments. A better understanding of the changes in the pelvic floor muscles, using a reliable method of assessment, could be helpful in providing proper care and improve quality of life for women after cancer. The aim of this study is to evaluate the impact of chemoradiation on the pelvic floor function at 4 and 9 months post-treatment in patients with CC.

## Materials and methods

This cohort study was conducted at the Barretos Cancer Hospital (BCH) from 2016 to 2018 and was approved by the Medical Research Ethics Committee of the Institution (Protocol Number: 1,477,063). Convenience sampling was used to prospectively select women aged between 20 and 70 years with CC, who underwent clinical staging, according to the International Federation of Gynecology and Obstetrics (FIGO) 2009 classification [16], from IB2 to IIIB and were treated with chemoradiation. Three-dimensional conformal external-beam radiation therapy (3D-CRT) was applied without image guidance, in 25 fractions of 1.8 Gy, totaling 45 Gy of external-beam radiation therapy dose, and in high dose brachytherapy (HDR); all women received 4 fractions with a mean dose of 27.69 Gy.

Chemotherapy was performed with cisplatin at a dose of 40 mg/m^2^ in 5 cycles a week in all women. All participants were informed about the study and signed an informed consent. The following were exclusion criteria from the study: women with any cognitive deficits that impaired the application of the tests, women who had undergone perineal (anterior and/or posterior perineoplasty) surgeries, women with peripheral neuropathy, women undergoing previous oncological treatments, women with conditions that prevented the introduction of vaginal probes, such as heavy bleeding or vaginal stenosis.

Eligible patients always had their pelvic floor strength evaluated by the same physiotherapist (TM), at three time points: Moment 1 (M1): before chemoradiation; Moment 2 (M2): at the first follow-up medical visit (usually 3 to 4 months after treatment); and Moment 3 (M3): at the second follow-up medical visit (usually after 6 to 9 months after treatment).

An initial interview was conducted with all eligible patients to fill out a questionnaire prepared by the researchers. Data collected included sociodemographic background and incontinence perceptions, along with a review of medical records for the collection of clinical data. Subsequently, assessments of the pelvic floor function were performed through perineometry (PNM) and surface electromyography (EMG). For evaluation of the pelvic floor, women were in a supine position, with hip and knee flexion. All women were correctly instructed to contract the pelvic floor by using digital palpation, and the verbal command for all assessments was to “perform the contraction as if they needed to hold the urine”.

PNM was performed using the Perina device (Quark Medical, ANVISA registration no. 80079190005), with a vaginal probe of dimensions 9.0 cm in length and 2.5 cm in diameter, lined with a non-lubricating condom. After the probe was properly positioned in the vaginal canal, three maximal voluntary contractions were requested, with a one-minute interval between them, to evaluate the phasic muscle fibers. After an interval of 3 minutes for rest, three contractions sustained for 6 seconds were requested, also with a one minute interval between them, in order to evaluate the tonic muscle fibers [17]. Duration of the intervals between these contractions was measured by means of a digital timer, and the average of the three contractions was used for the analysis.

For the evaluation of EMG, the Miotool 200/400 device (Miotec®) was used by way of the MiotecSuite software. A acquisition frequency of 2000 HZ was used for data collection; an amplifier gain of 2,000; common mode rejection rate (CMRR) of 110 dB; band-pass analog filter 20-500Hz of 4th order; Notch 60Hz filter and all its harmonics. To obtain the data, a Miotec® disposable intravaginal sensor was used, which was made of plastic and stainless steel contact bars of dimensions 85mmx25mm, lubricated with water-based gel. The reference electrode was positioned on the right lateral malleolus of the volunteer. A secondary sensor was also used in the abdominal muscle (right external oblique) to detect any synergism of this muscle with the pelvic floor. All surface electrodes used were positioned and fixed following the recommendations of the Surface Electromyography for the Non-Invasive Assessment of Muscles (SENIAM) [18]. Using this method three phasic contractions and three sustained tonic contractions were each requested for 6 seconds, with a range of 1 minute rest between contractions. Analysis were performed using mean contraction levels and peak normalization was obtained.

### Statistical analysis

The number and percentage of patients based on the frequency variables related to sexual intercourse and urinary incontinence were compared between the three time points (M1,M2, and M3) with the Cochran's Q test. Generalized Linear Models were used to study the evolution of the means of the pelvic floor strength at M1, M2 and M3 based on the measurement of PNM and EMG, and the longitudinal analysis to differences between mean categories for each variable in the stratified model. The Mann-Whitney test was used to compare the means of the categories at M1, M2 and M3, and Friedman test was used to do a intra-group analysis of the pelvic floor strength in each category. The normality of the data was verified by the Shapiro-Wilk test.

The data were collected through the REDCap [19] platform, and statistical analyses were performed using SPSS program (Statistical Package for the Social Sciences) version 21.0. Statistical significance was defined as *p*<0.05 and all *p-*values were two-sided.

## Results

**Fig 1:**
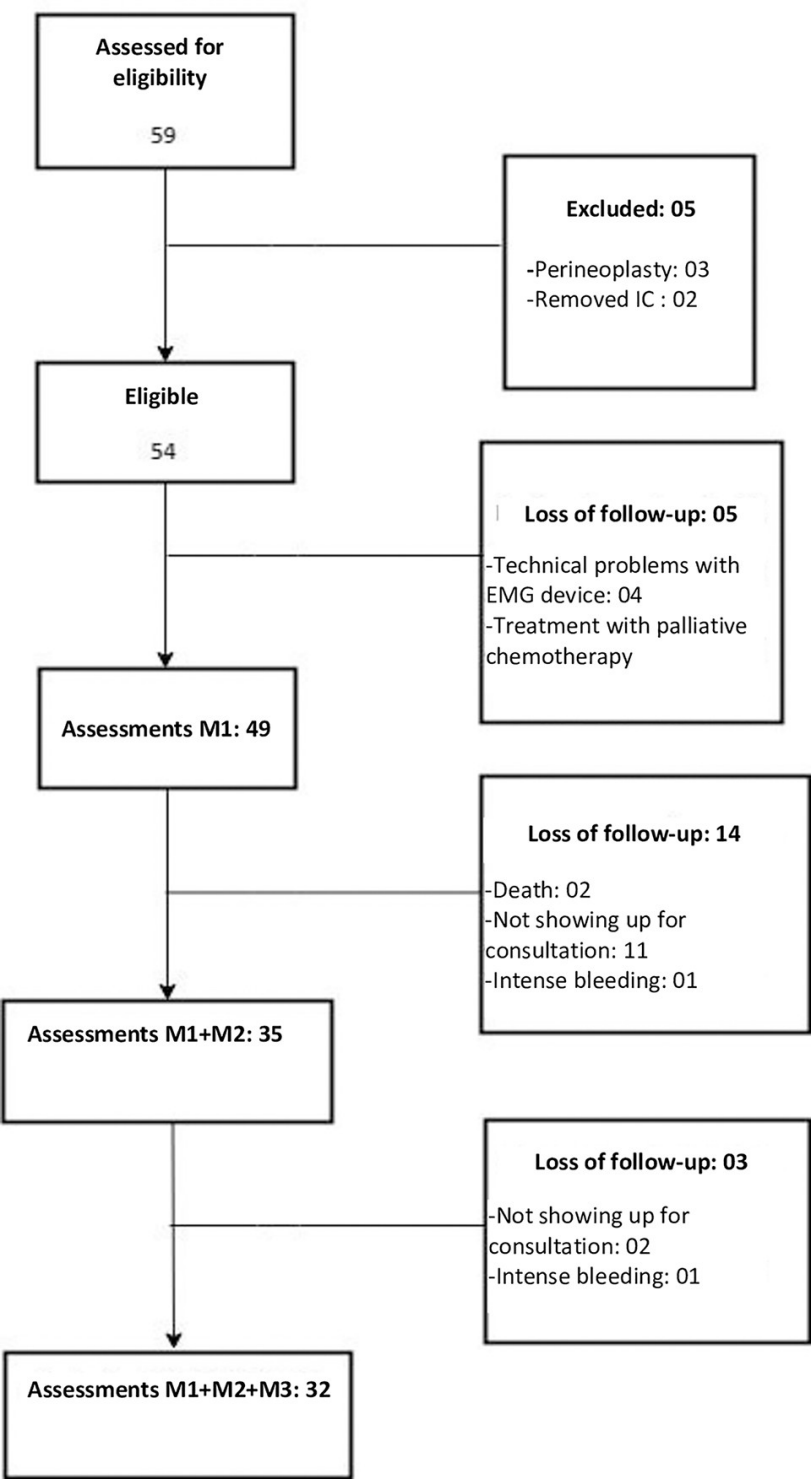
Recruitment flowchart and evaluation moments of study patients. IC: Informed Consent; EMG: Surface electromyography; M1: Evaluation at Moment 1 (before treatment); M2: Evaluation at Moment 2 (from 90 to 120 days after the end of the treatment); M3: Evaluation at Moment 3 (from 200 to 270 days after the end of the treatment.

We selected 59 patients who were treated with chemoradiation at BCH. Of these, 32 patients were evaluated and followed during the period from August 2016 to May 2018. Losses during follow-up and their reasons are detailed in Fig 1.

Statistical analyzes were performed with the 32 patients who were assessed at the three time points. Demographic and clinical characteristics are summarized in Table 1. The mean age was 47 (±11; 28–65) years, and the mean body mass index (BMI) 28.03(±6.63; 17.9–47) kg/m^2^ of the sample showed an overweight according to the classification of the World Health Organization [20]. Of the 32 women who underwent external-beam radiation therapy and HDR, 5 required an extra dose of external-beam radiation (boost).

**Table 1:** Demographic and clinical variables.

Demographic (n = 32)				
Mean age years (SD, min-max)	47	±11	28	65
Mean parity (SD, min-max)	3	±2	1	11
Mean BMI (SD, min-max)	28.03	±6.63	17.9	47
Menacme (n, %)	22	68.80%		
Menopause (n, %)	10	31.32%		
**Clinical**				
Staging (FIGO) (n, %)				
Ib2	2	6.20%		
IIa1	1	3.10%		
IIa2	0	0.00%		
IIb	19	59.40%		
IIIa	0	0.00%		
IIIb	10	31.20%		
Total mean external-beam radiation therapy dose (SD, min-max) (Gy)	45.0	±0.0	45.0	45.0
Total boost dose (SD, min-max)(Gy)	9	±0.0	9.0	9.0
Total dose HDR (SD, min-max)(Gy)	27.69	±1.03	24.0	28.0

Frequencies and percentages of patients according to sexual intercourse, stress and urgency urinary incontinence symptoms, and symptoms of fecal incontinence at different assessment points are summarized in Table 2.

**Table 2:** Number and percentage of patients according to the variables of sexual intercourse, urinary and fecal incontinence.

Variable	n	Time point evaluated	No		Yes		p*
Have you had sexual intercourse in the last 6 months?	32	M1	13	40.6%	19	59.4%	0.86
		M2	12	37.5%	20	62.5%	
		M3	13	40.6%	19	59.4%	
Is there a loss of urine in physical activities (sneezing, coughing, running)?	32	M1	18	56.2%	14	43.8%	0.12
		M2	23	71.4%	9	28.1%	
		M3	22	68.7%	10	31.2%	
Loss of urine occurs when there is a strong urge to urinate, difficult to control?	32	M1	27	84.4%	5	15.6%	**<0.001**
		M2	14	43.7%	18	56.3%	
		M3	18	56.2%	14	43.8%	
Is there involuntary loss of feces?	32	M1	32	100%	0	0.0%	0.61
		M2	31	96.9%	1	3.1%	
		M3	31	96.9%	1	3.1%	
Is there a will to defecate, difficult to control?	32	M1	32	100%	0	0.0%	
		M2	32	100%	0	0.0%	
		M3	30	93.7%	2	6.3%	

* Cochran's Q test.

There was no difference in sexual intercourse last 6 months (p = 0.86).

However, there were statistically significant differences in the symptoms of urgency urinary incontinence. The frequency of this symptom at M2 (56.3%) was higher than pretreatment (15.6%), (p<0.001). For the fecal incontinence symptom, there was no statistically significant difference between measurement at any of the time points (p = 0.61).

**Fig 2:**
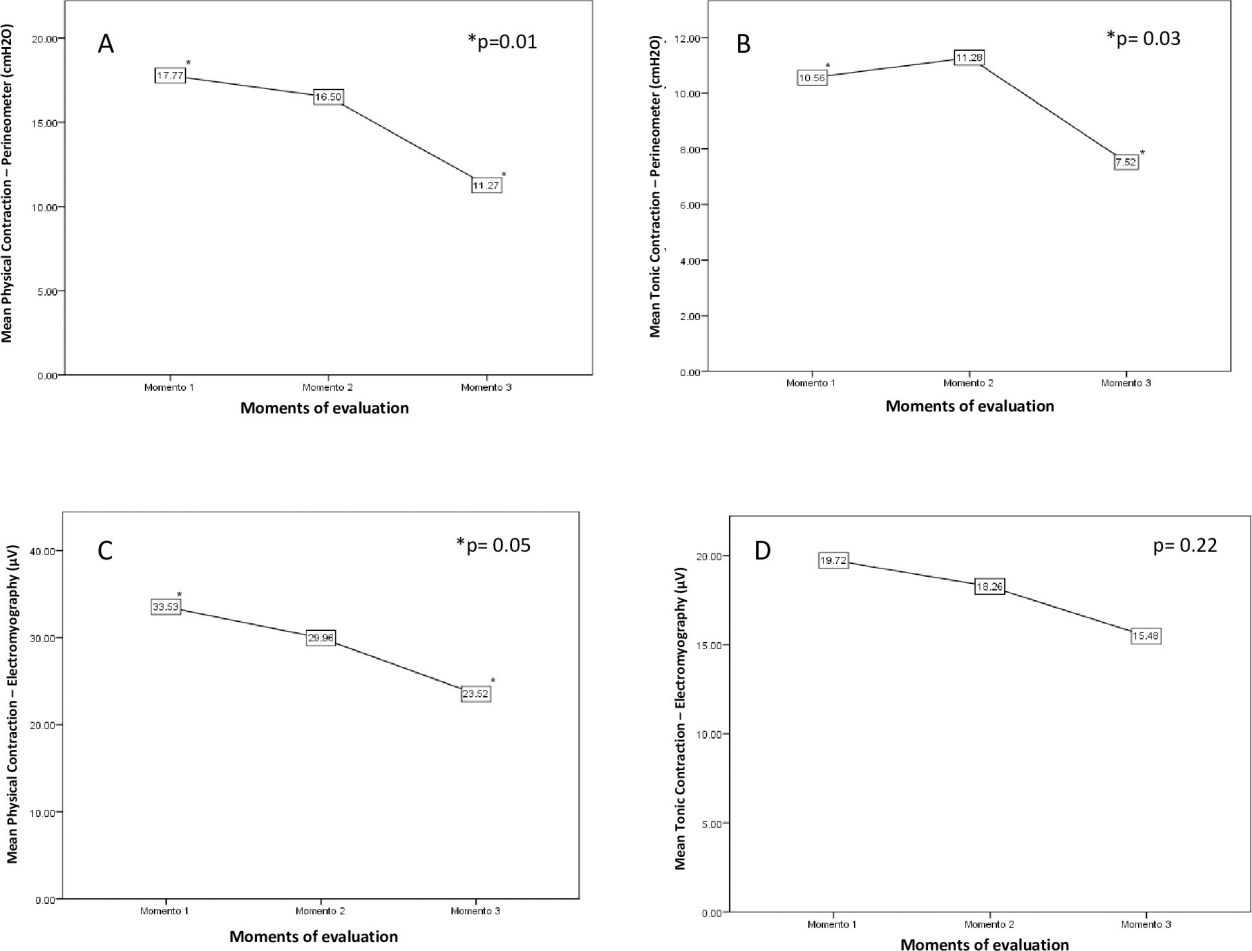
Means of phasic and tonic contractions measured through PNM and surface EMG, in the three moments of evaluation. A—Means of phasic contractions measured by PNM at moments of evaluation. B—Means of tonic contractions measured by PNM at moments of evaluation. C—Means of phasic contractions measured by EMG at moments of evaluation. D—Means of tonic contractions measured by EMG at moments of evaluation.

Regarding pelvic floor function over time (Fig 2), there was a reduction of the vaginal squeeze pressure on phasic and tonic contraction evaluated by PNM (p = 0.01 and p = 0.03 respectively). The means assessed at 9 months following treatment were lower compared to the means evaluated before treatment. For the means of electrical activity based on phasic contraction evaluated by EMG, there was a trend for a reduction in electromyographic activity at M3 in relation to before the treatment (p = 0.05).

The relationship between the means of muscle contraction in the phasic and tonic contractions evaluated by the two methods related to anthropometric variables, parity and hormonal status are described in the Tables 3, 4, 5 and 6. In the phasic contraction measured by PNM, obese women had a lower overall mean compared to non-obese women (p<0.001), and also had lower mean values at M1 and M2 evaluation time points(p<0.001 and p = 0.02 respectively), when means at all three time points were compared (Table 3) [16].

**Table 3:** Vaginal squeeze pressure (mmHg) in the phasic contraction evaluated by the perineometer related to anthropometric, parity and hormonal status.

			M 1 (n = 32)			M2 (n = 32)			M 3(n = 32)				
Variables	Categories	n	Mean	SD	p^1^	Mean	SD	p^1^	Mean	SD	p^1^	p^2^	p^3^
Age (years)	<50	17	16.63	2.29	0.65	15.02	2.29	0.37	10.55	2.29	0.35	0.21	0.07
	≥50	15	19.07	2.43		18.18	2.43		12.09	2.43			0.35
BMI (Kg/m2)	< 30	22	21.97	1.81	**<0.001**	18.67	1.81	**0.02**	12.79	1.81	0.07	**<0.001**	**0.00**
	≥ 30	10	8.53	2.69		11.73	2.69		7.93	2.69			0.58
Parity	0–3	25	18.99	1.87	0.34	17.28	1.87	0.75	11.01	1.87	0.5	0.25	**0.01**
	> 3	7	13.43	3.54		13.71	3.54		12.19	3.54			1.00
Hormonal status	Menacme	22	17.15	2.02	0.98	15.73	2.02	0.82	10.88	2.02	0.46	0.36	**0.04**
	Menopause	10	19.13	2.99		18.20	2.99		12.13	2.99			0.61

BMI: body mass index; p^1^: comparison of the means of the categories in each moment of evaluation; p^2^: comparison of the general means of the categories of each variable; p^3^: comparison of the means of each category along the evaluation moments.

**Table 4:** Vaginal squeeze pressure (mmHg) in the tonic contraction evaluated by the perineometer related to anthropometric, parity and hormonal status.

			M1 (n = 32)			M 2 (n = 32)			M3(n = 32)				
Variables	Categories	n	Mean	SD	p^1^	Mean	SD	p^1^	Mean	SD	p^1^	p^2^	p^3^
Age (years)	<50	17	9.10	1.50	0.55	10.88	1.50	0.37	7.41	1.50	0.76	0.26	0.12
	≥50	15	12.22	1.60		11.73	1.60		7.64	1.60			0.07
BMI (Kg/m2)	< 30	22	12.85	1.24	**<0.001**	11.24	1.25	0.20	8.36	1.25	**0.05**	**<0.001**	**0.01**
	≥ 30	10	5.53	1.85		11.43	2.36		5.67	2.36			0.38
Parity	0–3	25	11.09	1.25	0.37	11.02	1.33	0.47	7.63	1.34	0.65	0.66	**0.01**
	> 3	7	8.67	2.36		11.87	1.97		9.17	2.01			0.61
Hormonal status	Menacme	22	9.70	1.33	095	10.77	1.37	0.70	7.36	1.33	0.98	0.31	**0.04**
	Menopause	10	12.47	1.97		10.94	1.9		7.87	1.97			0.27

BMI: body mass index; p^1^: comparison of the means of the categories in each moment of evaluation; p^2^: comparison of the general means of the categories of each variable; p^3^: comparison of the means of each category along the evaluation moments.

**Table 5:** Pelvic floor muscle electromyographic activity (μV) in the phasic contraction evaluated by the EMG related to anthropometric, parity and hormonal status.

			M1 (n = 32)			M2 (n = 32)			M3(n = 32)				
Variables	Categories	n	Mean	SD	p^1^	Mean	SD	p^1^	Mean	SD	p^1^	p^2^	p^3^
Age (years)	<50	17	32.61	4.09	0.26	31.25	4.09	0.73	23.07	4.09	0.52	0.98	0.19
	≥50	15	34.57	4.35		28.50	4.35		24.03	4.35			0.42
BMI (Kg/m2)	< 30	22	31.91	3.55	0.82	32.78	3.55	0.17	24.16	3.55	0.76	0.59	0.28
	≥ 30	10	37.07	5.26		23.76	5.26		22.12	5.26			0.06
Parity	0–3	25	34.09	3.35	0.93	31.83	3.35	0.37	24.10	3.35	0.59	0.26	0.17
	> 3	7	31.52	6.33		23.29	6.33		21.45	6.33			0.56
Hormonal status	Menacme	22	30.14	3.53	**0.01**	29.23	3.53	0.79	21.86	3.53	0.06	0.09	0.18
	Menopause	10	40.97	5.24		31.58	5.24		27.18	5.24			0.15

EMG: surface electromyography; BMI: body mass index; p^1^: comparison of the means of the categories in each moment of evaluation; p^2^: comparison of the general means of the categories of each variable; p^3^: comparison of the means of each category along the evaluation moments.

**Table 6:** Pelvic floor muscle electromyographic activity (μV) in the tonic contraction evaluated by the EMG related to anthropometric, parity and hormonal status.

			M1 (n = 32)			M2 (n = 32)			M3(n = 32)				
Variables	Categories	n	Mean	SD	p^1^	Mean	SD	p^1^	Mean	SD	p^1^	p^2^	p^3^
Age (years)	<50	17	19.52	2.39	0.79	19.91	2.39	0.85	14.53	2.39	0.33	0.85	0.10
	≥50	15	19.96	2.54		16.38	2.54		16.55	2.54			0.24
BMI (Kg/ m2)	< 30	22	19.28	2.07	0.92	20.60	2.07	0.92	16.16	2.07	0.51	0.19	0.72
	≥ 30	10	20.69	3.06		13.09	3.06		13.98	3.06			0.20
Parity	0–3	25	19.80	1.98	0.79	18.95	1.98	0.82	15.18	1.98	0.39	0.76	0.18
	> 3	7	19.45	3.74		15.76	3.74		16.53	3.74			0.37
Hormonal status	Menacme	22	17.63	2.07	**0.03**	18.34	2.07	0.43	14.46	2.07	0.16	0.13	0.72
	Menopause	10	24.34	3.07		18.08	3.07		17.72	3.07			0.20

EMG: surface electromyography; BMI: body mass index; p^1^: comparison of the means of the categories in each moment of evaluation; p^2^: comparison of the general means of the categories of each variable; p^3^: comparison of the means of each category along the evaluation moments.

In the tonic contraction assessed by PNM, the BMI variable was also significant, in which obese women had a lower overall mean (p<0.001), and when mean values were compared at the moment of evaluation, obese women had lower mean values ​before treatment (p<0.001) (Table 4).

Mean values related to the variables age, BMI and parity did not have statistically significant difference in the phasic and tonic contraction evaluated by EMG (Tables 5 and 6). Only the hormonal status variable was statistically significant in the two contractions, where menopausal women had a higher mean in the evaluation before the treatment of phasic and tonic contraction (p = 0.01, p = 0.03 respectively).

Concerning intra-group analysis of the pelvic floor muscle strength, in both, phasic and tonic contraction by perineometer evaluation, we found that patients with BMI < 30 Kg/m^2^, number of pregnancy between 0–3 and hormonal status of menacme had loss of muscle strength from moment 1 to 3. This difference was not found with EMG evaluation.

## Discussion

A reduction in maximal vaginal squeeze pressure at Moment 3 in comparison to Moment 1 after chemoradiation for CC in phasic and tonic contractions assessed by PNM was observed. Changes in pelvic floor function before treatment was not demonstrated by EMG assessment, although there is a trend to reduce electrical activity in phasic contraction. Regarding the symptoms, there was an increase in urgency urinary incontinence, especially in the acute phase after chemoradiation, and we found that obesity, interfered with pelvic floor function.

In concordance with our findings, there are some authors that detected a relation between pelvic radiotherapy and pelvic floor dysfunction. Yeoh et al. [15], that evaluated pelvic floor function by rectal manometry, observed a reduction in the pelvic floor squeeze pressure following treatment with radiotherapy for CC. An analysis of the effects of radiotherapy after endometrial cancer by Bernard et al. [6] also found a reduction in maximal strength during a maximal voluntary contraction test. The authors also reported the loss of the ability to recruit motor units after radiotherapy within 1 to 5 years after treatment by assessing the pelvic floor muscle function by dynamometry [6].

However, in the study by Noronha et al. [13], which assessed the pelvic floor strength through bidigital palpation 6 months after treatment, there was no difference in the contraction strength of this musculature in women with CC after treatment with radiotherapy compared to women treated with hysterectomy.

One of the main factors that may contribute to the divergence of findings in literature is the different methods of evaluation employed, since the ways of measuring the function of this muscle are challenging due to its diaphragmatic shape and its connections to the organs and fascia of the pelvic region [8], and there is no consensus on the best method. Although there is no standard for the best way to evaluate, among all, the bidigital palpation is the least reliable method of evaluation that depends on the examiner's experience, therefore the preference in this study for reliable methods.

The tendency to the reduction of muscle strength after radiotherapy found in our study and in the literature, reinforces the deleterious effects of radiation on muscle fiber, as evidenced by an Oxford Based Evidence Medicine study, which shows with a level of evidence 2B that radiotherapy has effects on the structure of the pelvic floor between 2 and 62 months after radiotherapy in men with prostate cancer [21]. In addition to the changes in muscle fiber structure, one should also consider nerve modifications, which was suggested by Yeoh et al. [22] who found there was a lower muscle strength of the pelvic floor of men who received radiotherapy for prostate neoplasia, related to lesion of the pudendal nerve.

Another type of treatment used for its favorable anti-tumor properties was platinum-based chemotherapy. Despite the benefits, it can cause neurotoxicity by affecting large-diameter sensory nerve fibers, leading to a symmetrical glove and stocking type of sensory loss, numbness, tingling, pain, and burning sensation [23]. However, the drug does not seem to interfere with the pelvic floor function, as these symptoms are more related to peripheral neuropathy and become evident when there is a cumulative dose of the drug of 350 mg/m^2^ [23].

With the reduction of the pelvic floor strength after cancer treatment, related symptoms, such as incontinence, become more frequent [6, 11]. This study observed an increase in the frequency of patients with urinary urge incontinence symptoms. This finding was, especially evident in the acute phase after radiotherapy. In addition, we observed that there were some patients with incontinence and fecal urgency symptom after chemoradiation treatment, although the difference was not statistically significant. These results are compatible with previous literature, which has reported that after pelvic radiotherapy, there is an increase in the frequency of fecal urgency symptoms [15], a higher frequency in evacuation and diarrhea [13]. Another study found greater vesical and intestinal toxicity with a higher incidence of urgency symptoms and urinary frequency [6]. Other findings draw attention to a higher incidence of dysuria and fecal incontinence [11]. The study conducted by Oh et al. [24] supports the prior studies, regarding the effect of radiotherapy on vesical and bowel function. They evaluated the urodynamic effects of radiotherapy for CC treatment and demonstrated that radiotherapy decreased the maximum cystometric capacity and mean maximum flow but increased post-residual volume. Intestinal and vesical symptoms after radiotherapy are often present, and may be explained by the dose of radiotherapy applied to the puborectal muscle as well as to the internal and external sphincter muscles of the anus. Smeenk et al. [25] suggest that radiotherapy applied in these locations may contribute to the pathophysiology of incontinence and urgency.

In our study, the BMI variable interfered in the measurements of the pelvic floor strength that were evaluated by PNM in phasic and tonic contractions. In both contractions, the mean muscle strength of obese women was lower with non-obese women in both pretreatment and in the acute and chronic phase after treatment. Similar findings were found by Corrêa Neto et al. [26], who compared 26 obese women to non-obese women, and showed that the pressure strength of pelvic floor muscles evaluated by manometry was lower in obese women. In addition to lower muscle strength, 65.4% of the obese women had symptoms of fecal incontinence. Obesity, in addition to muscle weakness, is related to vesical symptoms, as it is thought to increases the intra-abdominal pressure, weakening pelvic floor muscles and thus predisposing to dysfunctions such as urinary incontinence [27]. In addition, the visceral adipose index is a useful item in the evaluation of incontinence risk, indicating the role of obesity in the pathophysiology of this dysfunction [28].

In this study, we observed that the variable hormonal status influenced the pelvic floor function, and that menopausal patients had a higher average muscle strength in the phasic contraction recorded by PNM and EGM and in the tonic contraction recorded by EMG. These results diverge from literature, which shows the influence of hormonal status on muscle structure where estrogen deficiency changes tissue structure due to lack of collagen, generating dysfunctions such as incontinence, sexual dysfunctions, and pelvic organ prolapse [29, 30]. Different results found in this study, compared to the literature, regarding hormonal status may be a reflection of the limitations of this study which is the small sample size. Other limitations are lost of follow-up during the study, short follow-up of 9 months after treatment and the lack of validated instruments to evaluate urinary and fecal symptoms.

It is important to point out that this study was designed to evaluate the influence of chemoradiation on the pelvic floor function. However, our study design is innovative because we used patients as their own internal control group by taking measurements before treatment, thus avoiding the biases of cohort comparison. In addition, we followed the evolution of this cohort over time, which is a methodology little used in the evaluation of the female pelvic floor after cancer treatment. Our analysis was able to demonstrate the effect of radiotherapy on the pelvic floor muscle function. Our findings suggest that in clinical practice, there is a need to perform follow-up evaluations of these patients by pelvic physiotherapy to ameliorate and prevent further symptoms. Future studies are needed to demonstrate the effects of radiotherapy on muscle tissue in the medium and long term with more objective methods such as magnetic resonance imaging.

## Conclusions

We observed that treating CC with chemoradiation influences pelvic floor muscle dysfunctions, and effects were more pronounced long after treatment. In addition, there was an increased frequency of urgency urinary incontinence symptoms. Our study also showed that characteristics of women such as obesity could interfere with muscle function unrelated to treatment.

## Supporting information

S1 File (XLSX)
